# Immigration allows population persistence and maintains genetic diversity despite an attempted experimental extinction

**DOI:** 10.1098/rsos.240557

**Published:** 2024-07-31

**Authors:** Keon Young Park, Mel Lucas, Andrew Chaulk, Stephen F. Matter, Jens Roland, Nusha Keyghobadi

**Affiliations:** ^1^ Department of Biology, Western University, London, Ontario N6A 5B7, Canada; ^2^ Department of Biology, Memorial University of Newfoundland, St John’s, Newfoundland A1C 5S7, Canada; ^3^ Department of Biological Sciences, University of Cincinnati, Cincinnati, OH 45221, USA; ^4^ Department of Biological Sciences, University of Alberta, Edmonton, Alberta T6G 2E9, Canada

**Keywords:** immigration, population rescue, genetic maintenance, genetic diversity, artificial removal, SNP

## Abstract

Widespread fragmentation and degradation of habitats make organisms increasingly vulnerable to declines in population size. Immigration is a key process potentially affecting the rescue and persistence of populations in the face of such pressures. Field research addressing severe demographic declines in the context of immigration among interconnected local populations is limited owing to difficulties in detecting such demographic events and the need for long-term monitoring of populations. In a 17-subpopulation metapopulation of the butterfly, *Parnassius smintheus*, all adults observed in two adjacent patches were removed over eight consecutive generations. Despite this severe and long-term reduction in survival and reproduction, the targeted populations did not go extinct. Here, we use genetic data to assess the role of immigration versus *in situ* reproduction in allowing the persistence of these populations. We genotyped 471 samples collected from the targeted populations throughout the removal experiment at 152 single nucleotide polymorphisms. We found no reduction in the genetic diversity of the targeted populations over time, but a decrease in the number of loci in Hardy–Weinberg equilibrium, consistent with a high level of immigration from multiple surrounding populations. Our results highlight the role of connectivity and movement in making metapopulations resilient to even severe and protracted localized population reductions.

## 1. Introduction

In the current age of widespread habitat loss and degradation resulting from anthropogenic activities, populations of many species across the globe are experiencing, or are vulnerable to, severe declines in size [1,2]. A severe decline in the number of reproducing individuals increases demographic stochasticity, reducing the likelihood of a population’s persistence, especially for organisms that depend on a threshold population number for successful breeding or survival (Allee effect) [3,4]. In addition, the loss of genetic diversity caused either by an initial or temporary reduction in population size (i.e. a bottleneck) or continued exposure to high levels of genetic drift can lead to inbreeding depression and reduced evolutionary potential of populations, exacerbating the risk of population extinction [5,6]. However, the effects of demographic and genetic stochasticity in small populations can potentially be countered through various forms of rescue, frequently involving the immigration of individuals into the population either naturally or through translocation by humans [7].

Three primary forms of population rescue are generally recognized: evolutionary, demographic and genetic. Evolutionary rescue is the recovery of a population by adaptation to the conditions that caused the initial decline [7,8]. In the strictest sense, evolutionary rescue occurs by natural selection acting on standing genetic variation and does not involve immigration from other populations [7,9]. However, a broader definition of evolutionary rescue can include selection acting on novel genetic variants introduced by immigrants that are particularly well suited to the altered or degraded environmental conditions [10,11]. Although more often reported under controlled laboratory conditions [12], examples of strict-sense evolutionary rescue do exist in nature, especially in organisms with rapid generation times, such as insects [13] and rodents [14]. Demographic rescue involves an influx of individuals through immigration that acts to directly augment population size and protect the population from stochastic demographic events and Allee effects [15,16]. Finally, genetic rescue refers to an increase in population fitness caused by the genetic contributions of immigrants that reduce the extent of inbreeding and inbreeding depression [17–19]. A broader definition of genetic rescue can include a role for genetic variation introduced by migrants in contributing to evolutionary potential, and therefore, there is some overlap in evolutionary and genetic rescue in their broadest senses [7]. Regardless, immigration probably plays a central role in many cases of population rescue [7,18], whether occurring through artificial translocation or natural dispersal.

Several studies demonstrate that connectivity among local populations, which increases the likelihood of between-population dispersal and immigration, is a key factor in the recovery or maintenance of local population numbers in the face of demographic crashes or bottlenecks [20–22]. Furthermore, populations rescued by immigration have shown increases in both genetic diversity and fitness [8,22,23]. An extreme example of genetic rescue is the rapid, albeit temporary, recovery of the isolated wolf population on Isle Royale, triggered by the dispersal of a single individual from the mainland population [24]. Overall, the positive effects of both demographic and genetic rescue have been established in various natural and experimental systems [18,25]. Furthermore, the importance of immigration to rescue processes and to the persistence of populations and metapopulations more generally, is widely recognized [26,27]. However, the ability of natural populations to persist under sustained conditions that severely elevate mortality or reduce reproduction and the potential for immigration to contribute to persistence under such conditions are less explored. It has been suggested that detailed, empirical assessments of the prevalence and power of immigration in maintaining populations lag behind our theoretical understanding [28].

Given the multitude of processes simultaneously contributing to rapid and extreme environmental changes across the globe, including climate change, land use change and invasive species, many populations may be confronted by sustained and severe pressures that reduce their abundance. It is, therefore, important to understand the key factors contributing to persistence in the face of such pressures to be able to inform management and conservation actions [29]. Here, we assess the role of immigration in allowing populations to persist despite a continuous and extreme elevation of mortality, along with reduction of reproductive output sustained over multiple generations. We genotype samples collected across an eight-generation-long experiment involving the continual removal of individuals from target populations within a natural metapopulation system of an alpine butterfly, the Rocky Mountain Apollo (*Parnassius smintheus*) [30].

**Figure 1.**
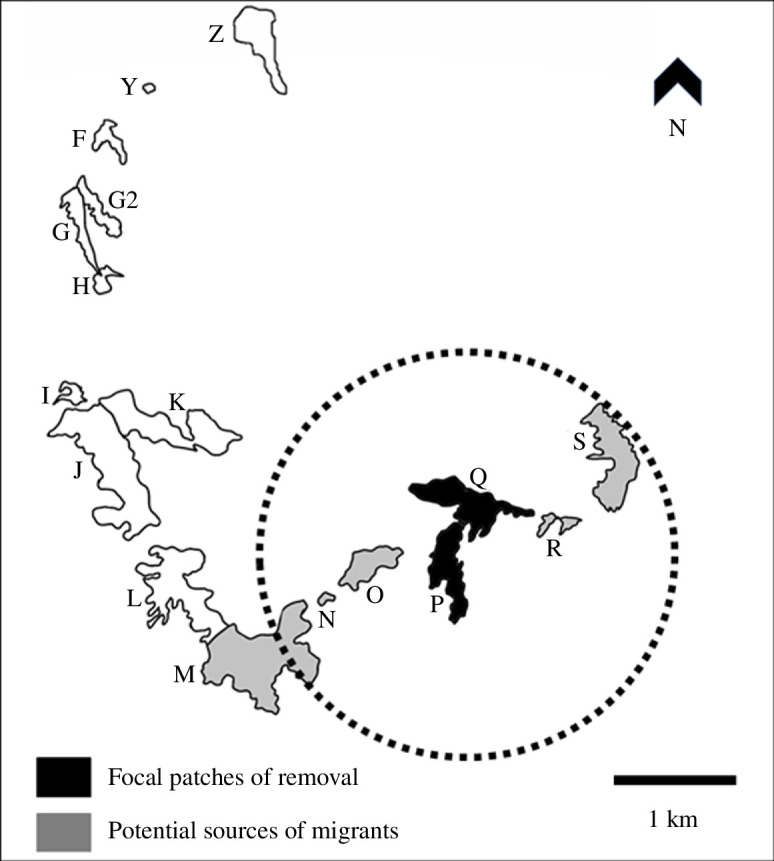
Map of the Jumpingpound Ridge study system located in Kananaskis Country, Alberta, Canada showing outlines of meadow habitat patches (above approximately 2000 m). Each habitat patch is labelled with a unique letter label. The dotted circle contains the section of the population network studied here, with patches P and Q (meadows filled in dark) being the target of artificial removals and other patches in the dotted circle (meadows filled in light grey) being within potential dispersal distance of the butterfly, *Parnassius smintheus* (i.e. within 2 km of patches P and Q).

The study metapopulation is located on Jumpingpound Ridge in the Rocky Mountains of Kananaskis Country, Alberta, Canada, where 17 subpopulations in alpine meadow habitat are separated by varying amounts of intervening forest [31]. Starting in 2001 and ending in 2008, the south-eastern portion of the network (figure 1) was subject to a long-term experiment in which all adults observed in two adjacent patches (P and Q, figure 1) were captured and removed each year, to examine effects of a simulated local extinction on the population dynamics of the surrounding patches [30,32,33]. Specifically, the aim of the removals was to mimic or induce an extinction event in the target patches, to test the hypothesis that their extinction would reduce immigration into neighbouring patches and thereby lead to reduced population sizes, increased extinction risk, and decreased growth in the surrounding populations proportional to their connectivity to the target patches [33]. While the removals aimed to induce local extinctions in patches P and Q, these target populations did not go extinct, nor did the likelihood of extinction in surrounding patches increase [30,33]. However, the reproductive output of the focal patches P and Q was reduced by more than 75% by the removals [30].

The populations in the target experimental patches persisted, therefore, despite the yearly removal of all observed adults over eight generations [30]. Two not mutually exclusive mechanisms may explain the survival of these populations. First, it is possible that not all adults in the two populations were removed each year and the offspring of those that were left behind experienced a higher per capita survival and reproductive success thanks to release from density-dependent factors [34]. Second, the removals may have created sinks that were recolonized by immigration from surrounding populations in the network. Here, we test these competing hypotheses by tracking genetic diversity of the target populations through the course of the experiment across the 8 years. If persistence occurred primarily through *in situ* reproduction from within the targeted populations, this would have created successive bottleneck events and we would therefore expect the local populations to decline significantly in genetic diversity over time [35,36]. Furthermore, we would expect a substantially greater reduction in allelic diversity relative to heterozygosity, as the former is more sensitive to bottleneck events [36]. However, to the extent that populations in the targeted patches P and Q persisted primarily owing to rescue through immigration from multiple different source populations, we would expect them to better maintain levels of genetic diversity despite the continuous removal of individuals. In this case, we would also predict decreasing Hardy–Weinberg equilibrium (HWE) over time owing to the continuous pooling of immigrants from genetically differentiated source populations (i.e. Wahlund effect) [37]. We, therefore, use genetic data collected over multiple generations to assess the relative roles of *in situ* reproduction versus immigration in allowing population persistence in the face of the attempted experimental extinctions.

## 2. Methods

### 2.1. Study organism, system and sample collection

The Rocky Mountain Apollo butterfly inhabits primarily high-altitude alpine meadow habitats throughout the Rocky Mountains in North America. The larvae’s main host plant is the perennial succulent, *Sedum lanceolatum* [38], though *Rhodiola integrifolia* is also occasionally used. The species is univoltine, completing a single generation each year. The larvae pupate after completing five instars, from which the adults emerge around July to August to mate and oviposit [39]. This annual adult flight season is the only time individuals can disperse from their natal habitat patch to another, as the larvae are not known to be able to move between patches. Dispersal is generally limited to neighbouring habitat patches; average recapture distances of marked adults within a flight season are only about 130 m for both males and females, and less than 10% of individuals are typically recaptured outside of the habitat patch in which they were originally captured and marked [31].

The Jumpingpound Ridge metapopulation occurs within a network of 17 patches of alpine meadow habitat ranging in size from 0.2 to 22.7 ha [31]. This system has been the subject of long-term population monitoring and genetic sample collection since 1995, with mark-recapture performed during the annual flight season to monitor population size and movement [31]. Populations in this system are genetically structured and normally display isolation by distance [40]. However, occasional network-wide bottlenecks in population size, thought to be driven by unfavourable over-wintering conditions, temporarily lead to increased genetic differentiation and loss of isolation by distance, as different alleles persist in different local populations; this differentiation declines rapidly following the bottleneck events and isolation by distance re-establishes as gene flow among the populations redistributes the surviving genetic variation [41,42]. The removal experiment on patches P and Q was conducted from 2001 to 2008 to investigate the effects of severe and continuous population reduction in patches P and Q on the population dynamics in surrounding, neighbouring patches [30,33]. Through the course of the experiment, patches P and Q were surveyed every 1–3 days during the flight season each year, and all observed butterflies were captured by hand netting and removed from the site. In total, 4830 butterflies were removed from P and Q over the 8 years. The combined number of individuals removed from both P and Q was highest in the first year of the experiment (approximately 1200) and lowest in 2003 (<100), a year in which the entire population network experienced a severe demographic bottleneck (fig. 2 in [30]). The removed individuals were labelled with the date and location of capture, and then stored, dried and pinned in a collection housed at the University of Cincinnati.

**Figure 2.**
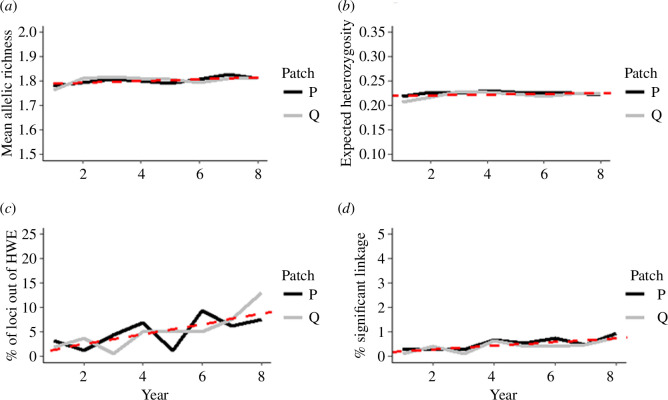
Changes in population genetic metrics over time within local populations in patches P (dark solid lines) and Q (light solid lines), through the course of an 8 year experiment in which all adults observed in each patch were removed each year from 2001 (year 1) until 2008 (year 8): (*a*) mean allelic richness, (*b*) expected heterozygosity, (*c*) percentage of single nucleotide polymorphism (SNP) loci not in HWE, and (*d*) percentage of SNP locus pairs in linkage disequilibrium. The dotted lines (red) represent the best-fit linear mixed model for each genetic response variable as a function of time (year of the experiment) as the fixed effect, with patch (P or Q) as a random effect.

We sampled from this collection of pinned specimens from patches P and Q, removing one leg per individual across each year of the removal experiment. For years in which the number of captured individuals in a patch was lower than 30, we sampled all available individuals. Otherwise, we sampled between 30 and 60 individuals per patch per year. Samples from 2001 represent the basal or initial state of the populations, as they would not yet have responded to the experimental manipulation. In addition, leg tissues from 42 frozen whole butterflies, removed during the 2008 flight season were also used; in combination with pinned samples from 2008, these represent the final year of the experimental removals. In total, we sampled 471 individuals from patches P and Q across 8 years (table 1). The forest separating patches P and Q forms only an incomplete barrier (figure 1) and individuals can move readily between these patches. To be consistent with previous studies in this system, including the removal experiments, we analysed samples from patches P and Q separately.

**Table 1.** Sample size and estimates of basic population genetic metrics (based on 152 polymorphic SNP loci) for samples from patches P and Q for each year of the removal experiment (2001–2008). (MAR, mean allelic richness; H_E_, expected heterozygosity; ‘loci out of HWE’, the proportion of loci significantly out of HWE.)

patch	year of collection	sample size	MAR	H_E_	loci out of HWE
P	2001	24	1.76	0.24	0.027
P	2002	20	1.75	0.21	0.011
P	2003	28	1.78	0.21	0.037
P	2004	29	1.77	0.22	0.059
P	2005	27	1.77	0.24	0.011
P	2006	30	1.77	0.24	0.080
P	2007	29	1.79	0.24	0.054
P	2008	43	1.78	0.24	0.064
Q	2001	21	1.71	0.22	0.021
Q	2002	30	1.77	0.20	0.037
Q	2003	14	1.78	0.22	0.005
Q	2004	31	1.79	0.22	0.043
Q	2005	28	1.78	0.24	0.043
Q	2006	26	1.76	0.23	0.043
Q	2007	30	1.79	0.24	0.064
Q	2008	61	1.78	0.24	0.123

To assess relationships of individuals removed from the target patches to populations that could have been potential sources of immigrants, we also genotyped samples from surrounding patches most likely to contribute migrants to P and Q. Based on *a priori* knowledge that individual *P. smintheus* travel approximately 130 m on average, to an observed maximum of 2 km within one flight season [31], we determined that the five neighbouring patches (M, N, O, R and S) within 2 km of P and Q contained potential source populations (figure 1). This is further substantiated by previous estimates of patch connectivity indicating that only patches M, O, R and S have sufficient connectivity to patches P and Q to be influenced by any changes in the population sizes of the latter [41]. Patch N is a small patch that harbours a very small local population (with population size typically in the single digits and subject to frequent extinction) [43]. Patch N is therefore an unlikely source of immigrants; furthermore, because of its low population size, no or very few samples are available from that patch in most years. While connected by movement of adults [31], these surrounding populations are semi-independent of the experimental populations, and of each other, as reflected in patterns of genetic differentiation [40–42] and a degree of asynchrony in their population dynamics [33].

Non-lethal samples of wing tissue were removed from individuals in surrounding populations during the course of mark-recapture studies, as described previously [31]. Briefly, a small (approximately 2 mm^2^) piece of wing tissue was removed from newly marked individuals and stored immediately in 100% ethanol until DNA extraction. With the exception of samples collected in 1995 [40], we did not have tissue samples available from the surrounding populations prior to 2005 as we were not regularly engaging in wing clip sampling before that time [42]. Here, we include genetic data from surrounding populations M, O, R and S, which are the only likely sources of direct immigrants to P and Q, in 2005 and 2008 (the final year of the experiment); these correspond to years in which genetic diversity and differentiation across the ridge were previously analysed using microsatellite data [42].

### 2.2. DNA extraction and single nucleotide polymorphism genotyping

We extracted DNA from leg and wing tissues using the DNeasy Blood and Tissue Kit (Qiagen, Germantown, MD). Each tissue sample was first homogenized in a microcentrifuge tube containing lysis buffer using a disposable pestle, before being incubated with proteinase K at 56°C for approximately 18 hours. The purified DNA was eluted from the Qiagen spin columns with 200 μl of purified water, preheated to 37°C. We genotyped each individual at a panel of single nucleotide polymorphism (SNP) loci using the Agena iPlex Gold MASSarray platform (Sequenom, San Diego, CA), which can perform robust genotyping assays using samples of low DNA concentrations (<0.01 ng μl^−1^).

Our SNP panel contained 171 unlinked SNP loci and was developed based on a double-digest restriction-site associated DNA sequencing (ddRADseq) dataset for the species [44]. Briefly, multiple ddRADseq libraries were constructed from 80 to 100 individuals each (for a total of 501 individuals, sampled from Jumpingpound Ridge and the surrounding region [45] and not overlapping with the individuals removed from patches P and Q) by digesting genomic DNA using the restriction enzymes *Nla*III and *Eco*RI, then selecting and amplifying fragments between 200 and 500 bp and sequencing the libraries on an Illumina HiSeq 2500 sequencer. We identified SNPs from the resulting sequences using the program STACKS [46] with the following parameters: minimum stack depth (*m*) = 3, mismatches allowed between putative catalogue loci (*n*) = 3, mismatches allowed between putative alleles (*M*) = 2, mismatches allowed to align secondary reads (*N*) = 4, and a maximum allowed missing data of 50%. This resulted in a ddRADSeq dataset of 8814 SNP loci. From the ddRADSeq sequences, we filtered SNPs with at least 40 bp upstream and downstream flanking sequences to allow space for designing primers for the iPlex Gold MASSarray assay, and used the software PLINK to identify and remove statistically linked loci. To identify SNPs that might putatively be functional, we aligned the filtered ddRADSeq sequences to a transcriptome of thorax tissue of adult butterflies captured during flight [47] using Magic-BLAST [48]. We defined fragments with at least a 90% sequence match as putatively expressed loci. Potential functions of these putatively expressed loci were identified using Trinotate [49], and 35 loci were chosen to reflect a variety of cellular functions such as metabolism, transcription regulation, transmembrane proteins, protein modification and insect development. An additional 130 loci having both less than 10% sequence match to the transcriptome and a greater than 90% sequence match to a shotgun-sequenced genome [50] were chosen at random to include in the final SNP panel. Finally, an additional six non-synonymous SNPs were included from the coding region of phosphoglucose isomerase (*Pgi*) [47], a gene associated with dispersal and flight metabolism in other insects, including the Glanville fritillary butterfly [51,52].

### 2.3. Genetic variation in the target populations over time

We estimated allele and genotype frequencies, and metrics of genetic diversity and differentiation, in each year of the removal experiment, separately for patches P and Q. All analyses were performed in the R statistical platform [53].

#### 2.3.1. Genetic diversity

We estimated allelic richness, rarefied to 14 individuals (the lowest sample size, in patch Q in year 3), using the package ‘PopGenReport’ [54] and conducted a Wilcoxon test for the significant differences in mean allelic richness (averaged across loci) between year 1 of the experiment (i.e. original population unaffected by the yearly removals) and year 8 (final year of removals). We estimated expected heterozygosity and tested for a significant difference in mean expected heterozygosity across loci between the first and last year of the experiment using the package ‘adegenet’ [55]. We also fitted each of the mean allelic richness and mean expected heterozygosity in a separate linear mixed model in the ‘nlme’ package [56] with year as a numeric predictor (i.e. reflecting time since the start of the removal experiment) and patch identity (P or Q) as a random factor.

#### 2.3.2. Hardy–Weinberg and linkage

We tested for HWE at each locus in each year using the package ‘pegas’ [57], with the number of Monte Carlo replicates set to 1000 and *α* = 0.01 to determine significance. Linkage disequilibrium tests were conducted for each pair of loci in each year with Genepop 4.7.5 [58], with the number of dememorizations set to 10 000 and α = 0.01 to determine significance. We fitted the proportion of SNPs out of HWE and the proportion of locus pairs in linkage disequilibrium in separate linear mixed models in the ‘nlme’ package [56] with year as a numeric predictor (i.e. reflecting time since the start of the removal experiment) and with patch identity (P or Q) as a random factor.

#### 2.3.3. Genotype and allele frequency changes across the removal experiment

To capture changes in allele frequency distributions across all loci over time, we used the R package ‘hierfstat’ [59] to estimate pairwise *F*_ST_ between samples from consecutive years of the experiment (i.e. year 1–2, 2–3, etc.), as well as between year 1 and each subsequent year (i.e. year 1–2, 1–3, etc.), within each of patches P and Q.

To test for changes in genotype frequencies of individual loci over time within each of patches P and Q we used a series of multinomial logit models, implemented in R using the package ‘mclogit’ [60]. We coded genotypes at each locus in a trinomial form, with the minor (less common) homozygote, heterozygote and the major (more common) homozygote assigned a value of ‘0’, ‘1’ and ‘2’ respectively. Owing to the trinomial form of the multinomial model, all assayed SNPs that were monomorphic or lacked the minor (less common) allele homozygote in the dataset were also removed, such that we assessed changes in genotype frequencies at 145 SNPs in patch P and 135 SNPs in patch Q. The observations of each of the three possible genotypes were then modelled as a function of time (years), separately for each SNP and in each patch. Significance values were adjusted for multiple tests using the Benjamini–Hochberg correction [61].

### 2.4. Relationships to putative sources of immigrants

#### 2.4.1. Pairwise *F*_ST_ relative to surrounding patches

We estimated pairwise *F*_ST_ between populations in each of the experimental patches (P, Q) and each of the surrounding patches that are potential sources of immigrants (M, O, R and S) in 2005 (the first year of the experiment for which genotype data were available for surrounding patches) and 2008 (the final year for the experiment) using the ‘hierfstat’ package [59].

#### 2.4.2. Population assignment of potential dispersers

We used the approach implemented in Geneclass 2.0 [62], with the Rannala & Mountain [63] Bayesian method of assignment, to assign individuals removed from patches P and Q to putative patches of origin from the set of neighbouring, potential source populations. Importantly, these assignment analyses were not intended to test or validate immigration as the main factor allowing the persistence of populations P and Q. Instead, once we had inferred a primary role of immigration through temporal changes in genetic diversity and HWE, the assignment tests were conducted to examine potential sources of those immigrants. In particular, our goal was to assess which neighbouring patches provided the majority of immigrants, and if the source of immigrants was stable or variable over time. These analyses make the assumption that all individuals captured and removed from patches P and Q are first-generation migrants from surrounding patches; while some removed individuals may have been born in P and Q, our analysis of genetic diversity over time suggested that a large proportion removed each year were likely to be first-generation immigrants. We only assigned individuals removed from P and Q in 2005 and 2008, years in which genotype data from neighbouring populations were also available, and we only considered assignments to surrounding populations using data from the same year (i.e. samples removed in 2005 from P and Q were assigned to surrounding populations using 2005 data from those populations). To assess the quality of assignments provided by our SNP dataset, we also assigned all individuals sampled in the potential surrounding source populations to the same set of populations (M, O, R and S), separately for 2005 and 2008; the extent to which individuals are assigned back to their ‘own’ population provides an index of the quality of assignments in a given dataset [62].

## 3. Results

### 3.1. Genetic variation in the target populations over time

Of the 171 SNPs targeted for genotyping, assays for three SNPs failed altogether, seven SNPs failed across more than 70% of samples, and nine SNPs were monomorphic across all samples in all years from both patches P and Q. These 19 SNPs were removed, and we used the genotype data from the remaining 152 polymorphic SNPs for further analyses. Of the 477 individuals genotyped from P and Q, six individuals were successfully genotyped at less than 80% of all SNPs and were also removed from the dataset. The total genotype failure rate across all remaining 471 individuals and 152 SNP loci used for further analyses was 4.1%.

#### 3.1.1. Genetic diversity

Populations in both patches P and Q showed a slightly increasing trend in mean allelic richness over the course of the removal experiment, with only minor oscillations (figure 2*a*). The mean allelic richness in a given year ranged from 1.764 to 1.827 (s.e. **=** 0.005) for patch P and 1.764 to 1.816 (s.e. = 0.005) for patch Q. A Wilcoxon test comparing the first and final years of the experiment (i.e. years 1 and 8) indicated no significant change in mean allelic richness through the course of the entire experiment for either P (*p* = 0.359) or Q (*p* = 0.543). However, the year was a significant predictor of mean allelic richness across the entire dataset with a positive slope coefficient indicating a trend of increasing allelic richness over time (*p* = 0.023, *β* = 0.004 ± 0.001; figure 2*a*).

The mean expected heterozygosity was also mostly stable throughout the experiment with a slightly increasing trend (figure 2*b*). The mean expected heterozygosity in a given year during the experiment ranged from 0.219 to 0.230 (s.e. **=** 0.001) for patch P, and 0.207 to 0.229 (s.e. **=** 0.002) for patch Q. Testing for a significant difference between the expected heterozygosity of the first and final years (i.e. years 1 and 8) of the experiment, we found no significant difference in patch P (*p* = 0.798) but did find one in Q (*p* = 0.014), where the mean expected heterozygosity increased from 0.207 in year 1 to 0.224 in year 8. The year was not a significant predictor of mean expected heterozygosity across the entire dataset (*p* = 0.209, *β* = 0.76 × 10^–3^ ± 0.57 × 10^–3^).

#### 3.1.2. Hardy–Weinberg and linkage

The proportion of SNPs out of HWE in each patch fluctuated throughout the experiment but showed a strong increasing trend towards the end of the experiment, particularly in patch Q (table 1; figure 2 *c*). In the initial year of the experiment (i.e. representing the ‘pre-removal’ conditions), 3.11% and 1.86% of loci were out of HWE in patches P and Q, respectively; by the final year, these values increased to 7.45% and 13.04%, respectively. The year was a significant positive predictor of the number of loci out of HWE overall (*p* = 0.001, *β* = 1.040 ± 0.249).

The proportion of SNP pairs in linkage disequilibrium also fluctuated over time but displayed a strong increasing trend towards the end of the experiment in both patches P and Q (figure 2*d*). In the initial year of the experiment (i.e. representing the ‘pre-removal’ conditions), approximately 0.028% and 0.009% of pairs of loci were in linkage disequilibrium in patches P and Q, respectively; by the final year, these values increased by approximately three and ninefold to 0.095% and 0.077%, respectively. Year was a significant positive predictor of the number of pairs of loci in linkage disequilibrium (*p* < 0.001, *β* = 0.756 × 10^−3^ ± 0.0001).

#### 3.1.3. Genotype and allele frequency changes across the removal experiment

Year-to-year (i.e. year 1–2, 2–3, etc.) pairwise *F*_ST_ estimates were low to moderate in both patches, ranging between 0.003 and 0.012 (mean = 0.007 ± 0.001) in patch P, and between 0.003 and 0.01 (mean = 0.006 ± 0.0008) in patch Q. Pairwise *F*_ST_ estimates between year 1 and each subsequent year of the experiment were similarly low, ranging from −0.004 to 0.008 (mean = 0.002 ± 0.0014) in patch P, and from −0.002 to 0.005 (mean = 0.002 ± 0.0008) in patch Q (electronic supplementary material, figure S1). Pairwise *F*_ST_ estimates between years 1 and 8, the first and final years of the study, were also low (P: *F*_ST_ = −0.007; Q: *F*_ST_ = −0.005). None of the pairwise *F*_ST_ estimates, including comparisons between the first and final years of the experiment, were significantly different from zero (i.e. the 95% confidence intervals (CIs) bracketed zero), with the exception of the comparison between years 1 and 6 in patch P (*F*_ST_ = 0.008; 95% CI = 0.0008–0.017).

After controlling for a false discovery rate, we did not detect any SNPs that showed significant change in genotype frequency across years of the experimental removals.

### 3.2. Relationships to putative sources of immigrants

#### 3.2.1. Pairwise *F*_ST_ relative to surrounding patches

Pairwise *F*_ST_ between each of the experimental patches (P, Q) and immediately surrounding patches (M, O, R and S) were low to moderate within each year tested and declined in value overall from 2005 to 2008. In 2005, *F*_ST_ estimates ranged between 0.038 and 0.079 for all pairwise comparisons involving patch P (mean = 0.050 ± 0.010) and between 0.037 and 0.074 for all pair-wise comparisons involving patch Q (mean = 0.050 ± 0.008). In 2008, the *F*_ST_ values ranged between 0.026 to 0.032 for all pairwise comparisons involving patch P (mean = 0.030 ± 0.001) and between 0.026 to 0.031 for all pairwise comparisons involving patch Q (mean = 0.030 ± 0.001). Differentiation among populations in the non-experimental patches themselves (M, O, R and S) declined from 2005 to 2008, with mean pairwise *F*_ST_ among only those populations at 0.046 ± 0.016 in 2005 and 0.006 ± 0.002 in 2008.

#### 3.2.2. Population assignment of potential dispersers

Individuals removed from the experimental patches could be assigned to a given neighbouring patch with generally high confidence. The assignment score (likelihood of a given population as the source of the individual relative to the sum of likelihoods for all populations, expressed as a percentage; [62]) to the most likely potential source population, averaged among all individuals collected from P and Q (in both 2005 and 2008), was 84.29** ±** 2.26% and 82.47 ± 1.61%, respectively, compared with 14.51 ± 1.96% and 13.33 ± 1.30% for the second most likely source population. For the samples collected from the surrounding patches M, O, R and S, all individuals in 2005 and all but two individuals in 2008 were assigned to their 'own' patch with the highest likelihood (in 2008, one individual sampled in O was assigned to M with an assignment score of 95.5% and one individual sampled in S assigned to R with an assignment score of 75.7%). The mean assignment score for individuals from the surrounding patches to their own patch (i.e. where they were sampled), which provides an index of the quality of the assignments [62]), was 97.5% in 2005 and 95.1% in 2008.

**Figure 3.**
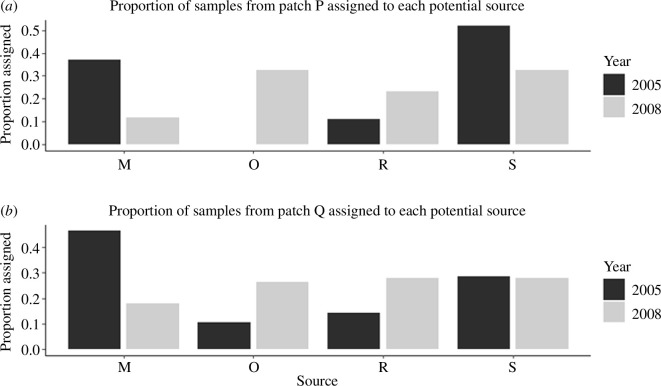
Proportion of samples collected in (*a*) patch P, and (*b*) patch Q, in the 2005 (dark bars) and 2008 (light bars) flight seasons, assigned to each of four surrounding populations that are potential sources of immigrants (in neighbouring patches M, O, R and S, located within 2 km of the target patches). Each individual removed from patch P or Q was assigned to the most likely of the potential source populations based on genotypes at 152 polymorphic SNP loci, using the Rannala & Mountain [63] Bayesian method of assignment implemented in Geneclass 2.0.

In 2005, the majority of individuals removed from patch P were assigned with the highest likelihood to neighbouring patches M (37.0% of individuals) and S (51.9% of individuals), with 11.1% assigned to R and no individuals assigned to patch O (out of 27 individuals sampled from P in 2005). However, the relative contribution of inferred immigrants to patch P from patches M and S declined in 2008 (proportion of sampled individuals assigned with the highest likelihood to M: 11.6%, and to S: 32.6%), while patch O and R’s contributions increased substantially with 32.6% and 23.3% of individuals assigned, respectively (figure 3).

About half of the individuals removed from patch Q in 2005 were assigned with the highest likelihood to patch M (46.4% of individuals). In 2005, only one individual captured in patch Q was assigned with the highest likelihood to O, out of 28 total individuals. However, patch Q also displayed a large apparent decrease in inferred immigration from patch M, and an increase in immigration from patch O, from 2005 to 2008. The proportion of individuals captured in Q that assigned with highest likelihood to patch M more than halved from 2005 to 2008, while those assigned to patch O increased by almost 20% points, making the inferred contribution of immigrants to experimental patch Q fairly even across the source populations in 2008, compared with 2005. (figure 3).

## 4. Discussion

The continual removal of all observed adult individuals from two populations over eight consecutive generations did not result in the extinction of those populations [30,33]; we show here that those removals also did not lead to a reduction of genetic diversity in the populations. Our results support immigration from neighbouring populations as the main process allowing the persistence despite the experimental removals and highlight the resilience of this metapopulation system to severe, localized reductions in survival and reproduction.

Any isolated population experiencing the high level of mortality and reduced reproductive output induced by the experimental removals consistently over several generations [30], would be expected to show a significant decline in genetic diversity [34,64]. If such an isolated population was not driven to extinction and persisted primarily through *in situ* reproduction, the resulting bottleneck events over generations would cause a significant decline in allelic diversity, in particular [36,65]. However, mean allelic richness and expected heterozygosity for both experimental patches P and Q did not decline across the years, with allelic richness even showing a moderate but significantly increasing trend over time (figure 2*a*,*b*). The maintenance of genetic diversity, especially allelic richness, supports the alternative mechanism for the persistence of these populations: consistent demographic recovery by incoming dispersal from other populations in the network.

Further supporting the immigration hypothesis is the significant increase in the proportion of loci out of HWE within the experimental populations over time (figure 2*c*); a continuous, yearly influx and pooling of dispersers from multiple partially differentiated source populations would be expected to lead to increasing deviations from HWE through the Wahlund effect [37]. By contrast, successive bottlenecks would not be expected to lead to increasing deviation from HWE (i.e. a mismatch between observed allele and genotype frequencies) with each generation, as long as the probability of an individual being removed from the population was random with respect to genotype and matings among surviving adults were also largely random. Consistent with an increasing number of loci out of HWE, we also observed a substantial increase over time in the numbers of pairs of loci in linkage disequilibrium (figure 2*d*), although this pattern would be expected under both hypotheses of mixing of immigrants from multiple gene pools and successive bottlenecks [66].

Additional evidence in support of the immigration hypothesis is the lack of differentiation of successive generations in each of the experimental patches from their starting state (i.e. *F*_ST_ not significantly >0 between even the first and final year of the experimental removals). In the absence of significant immigration, we would expect successive local bottlenecks to lead to rapidly increasing genetic distance between the initial population state and each subsequent generation. By contrast, we would expect a continual source of immigrants from a given set of surrounding populations to result in much more stable allele frequencies over time, as was observed here, with perhaps an initial increase in genetic distance only after the first year of removals.

We also observed a decline in genetic differentiation over time (i.e. in 2005 versus 2008) between each of the populations in P and Q and their surrounding populations. This pattern is concordant with a previously described decline in genetic differentiation among all populations on the ridge over the same time interval, associated with synchronous fluctuations in population size [41,42]. Specifically, the entire ridge experienced a severe collapse in adult population numbers in 2003, followed by a recovery of population sizes over the following years. Previous work has shown that genetic differentiation among populations on the ridge increased after the initial collapse (as measured in 2005) as different alleles survived in different local populations, but rapidly declined over subsequent years (as measured in 2008) through gene flow [41,42]. The increased genetic similarity between each of P and Q and their surrounding populations between 2005 and 2008 may partly reflect the changes in genetic differentiation across all the non-experimental populations on the ridge as result of population cycles. Importantly, however, the differentiation between each of P and Q and their surrounding populations declined only slightly from 2005 to 2008 (by approximately 40% on average), while the differentiation among all the surrounding populations to each other declined considerably more (almost eightfold; electronic supplementary material, table S1). Therefore, although allele frequencies in the surrounding populations (and relationships of those populations to each other) changed considerably from 2005 to 2008, the relationships between each of P and Q to those surrounding populations were much more stable over the same time. This observation is also consistent with a hypothesis of high levels of sustained immigration over that period from all the surrounding populations into patches P and Q.

Importantly, we do not suggest that no individuals were left behind in patches P and Q after the yearly removals or that there was zero *in situ* reproduction. Indeed, it is very likely that at least some adults present in the populations were left behind each year of the experiment and that they did reproduce locally. However, our results do indicate that immigration from surrounding populations was the dominant process, relative to *in situ* reproduction and population growth, that allowed populations to persist and maintain the sizes of those populations through the experiment.

There are several examples of populations showing no or minimal loss of genetic diversity following observed demographic bottlenecks as a result of immigration from external sources [20,21,67]. Furthermore, previous work on *P. smintheus* in the Jumpingpound Ridge system found that patch connectivity, which facilitates immigration, was strongly associated with faster recovery of allelic diversity within local patches after the ridge-wide demographic collapse in 2010 [68]. Our current study highlights the role of immigration in maintaining genetic diversity and allowing population persistence in the face of even extreme and lengthy demographic pressures, further supporting the importance of patch connectivity for both demographic and genetic resilience. Although other studies have demonstrated the ability of immigration to counter high local mortality in populations, including from lethal culling of managed populations [69,70], our study represents an extreme case of severe population reduction (all observed adults removed from the population) occurring long term (over eight consecutive generations), and represents a particularly strong test of the ability of immigration to maintain local populations.

With immigration implicated as the main driver of population persistence in patches P and Q across the experiment, we investigated the relative contributions of potential source populations to the immigrant pool using genetic assignment tests. The assignment tests suggested some degree of immigration from all potential sources, consistent with the maintenance of genetic diversity in patches P and Q and with the increase in numbers of loci out of HWE (which may be ascribed to a Wahlund effect), over time. Therefore, immigration into the target patches appeared to follow largely a migrant pool model with the mixing of individuals from multiple potential sources, as opposed to a propagule pool model with migration from a single source [71].

On the one hand, minimal genetic differentiation between samples from consecutive years in patches P and Q (i.e. non-significant temporal *F*_ST_ estimates) suggests a relatively stable pool of potential immigrants over time (electronic supplementary material, figure S1). Nonetheless, the genetic assignment of individuals captured in patches P and Q in 2005 and 2008 indicated potentially subtle variation in the source of migrants. Specifically, we observed a decrease in the relative contribution of inferred dispersers from patch M to both patches P and Q between those years, and an increase in the contribution from patch O. Patches R and S showed a relatively stable contribution of immigrants between 2005 and 2008, with their contribution into patch P and Q changing only moderately between these years. Patch M is the largest in the spatial network and, among the potential source patches for P and Q, contains the largest population of *P. smintheus* in most years [43]. Interestingly, despite the decline in patch M’s inferred contribution of immigrants to patches P and Q from 2005 to 2008, population size in M itself did not decline in that time period based on mark-recapture estimates [43,68]. In fact, in 2008 the largest population size estimate for M over the last two decades was observed, with the estimated local population size more than triple that estimated in 2005 [43]. Negative density-dependent dispersal has been observed in this species, with individuals more likely to emigrate from less dense populations and less likely to leave more dense populations [31]; this effect may explain decreased immigration from patch M in the year in which population size there was larger. Regardless, the assignment test results support the hypothesis that the persistence of populations in patches P and Q was not dependent on a single primary source population, but rather on the broader network with multiple sources contributing to the yearly pool of immigrants.

We recognize important caveats and assumptions in our assignment of individuals to potential source populations. First, we are assuming that all sampled individuals (i.e. all individuals removed from the experimental populations) are immigrants. Given the important role of immigration suggested by our analyses of genetic diversity and HWE over time, most individuals are indeed likely to be immigrants or direct descendants of immigrants; nonetheless, at least some adults removed from the experimental population may have been born locally. Second, the metapopulation is characterized by isolation-by-distance and adjacent populations are genetically similar [40,42]. This could limit our power to accurately assign individuals between adjacent potential source populations; we may have more confidence in assigning individuals generally to one side or the other of the targeted, experimental populations (i.e. M and O versus R and S) as those sets of populations are more strongly differentiated. Given these caveats, we do not interpret any individual assignment as unequivocally representing a specific immigration event. Instead, our aim was to assess general trends in immigration to the experimental patches over space and time, on aggregate and overall inferred assignments.

The increased representation of immigrants within P and Q in response to the experimental removals could potentially have been associated with selection for traits that facilitate immigration or colonization. For example, in a metapopulation of the Glanville fritillary butterfly (*Melitaea cinxia*), more isolated local populations show significantly higher frequencies of alleles at the *Pgi* locus associated with flight metabolism and ability compared to less isolated populations, suggesting selection for these alleles during dispersal or colonization [72,73]. We did not detect significant directional changes in the frequencies of genotypes at any individual loci in response to the experimental removals in patches P and Q, and therefore no evidence of selection in response to either the removal itself or subsequent immigration; however, this result is not surprising considering the moderate size of our SNP panel.

We present evidence for demographic rescue facilitated by immigration in response to a long-term, multi-generation artificial removal experiment. Interestingly, although loss of immigrants from the target patches P and Q to surrounding populations because of the experimental removals had little impact on the persistence of those surrounding populations [30,33], our results here indicate that immigration into patches P and Q, by contrast, was probably a key factor buffering those experimental populations against extinction. The lack of an effect of removals from P and Q on the persistence of surrounding populations may partially reflect ongoing movement and connectivity among the surrounding populations. Our results provide further evidence for the resilience of this metapopulation to both local and network-wide demographic decline [41,68] and highlight the importance of movement and connectivity for the persistence of organisms existing in fragmented landscapes [74].

## Data Availability

A copy of the SNP dataset is available on Dryad [75]. Supplementary material is available online [76].
